# Systemic Medication Adjustment and Short-Term Relapse of Cytomegalovirus Retinitis After Allogeneic Hematopoietic Stem Cell Transplantation: A Research Communication

**DOI:** 10.3390/biomedicines14102189

**Published:** 2026-09-28

**Authors:** Shuo Wu, Yihong Ding, Jing Hou

**Affiliations:** 1 Department of Ophthalmology, Peking University People’s Hospital, Beijing 100044, China; 2 Beijing Key Laboratory of Ocular Disease and Optometry Science, Peking University People’s Hospital, Beijing 100044, China

**Keywords:** cytomegalovirus retinitis, allogeneic hematopoietic stem cell transplantation, medication adjustment, relapse, interleukin-8, Cox regression

## Abstract

**Background**: Cytomegalovirus retinitis (CMVR) remains a sight-threatening complication after allogeneic hematopoietic stem cell transplantation (allo-HSCT). Systemic antiviral, immunosuppressive, and anti-infective regimens are often modified because of transplant-related complications, but the association between these modifications and ocular relapse remains unclear. **Methods**: We analyzed a prospective cohort of 44 patients with CMVR after allo-HSCT (65 affected eyes) who were followed for three months after discontinuation of local antiviral therapy. The primary analysis used Cox proportional hazards regression at the eye level with robust standard errors clustered by patient. Exact dates of the first systemic medication adjustment were retrieved, and additional time-dependent Cox and patient-level time-to-first-relapse Cox analyses were performed. **Results**: During follow-up, 24 eyes from 19 patients relapsed, and 18 patients underwent systemic medication adjustment. The first adjustment occurred a median of 25.5 days after discontinuation of local therapy (range, 7 to 72 days). Among the 13 adjusted patients who relapsed, all first adjustments preceded relapse, with a median adjustment-to-relapse interval of 15 days (range, 3 to 52 days). In the primary model, medication adjustment was associated with relapse (HR, 8.254; 95% CI, 2.656 to 25.645; *p* < 0.001), as was baseline aqueous IL-8 (HR, 1.190 per 100 pg/mL; 95% CI, 1.088 to 1.301; *p* < 0.001). In the time-dependent eye-level analysis, medication adjustment remained associated with relapses (HR, 11.360; 95% CI, 4.330 to 29.803; *p* < 0.001). A patient-level time-to-first-relapse Cox analysis showed a similar association (HR, 4.477; 95% CI, 1.688 to 11.878; *p* = 0.003). **Conclusions**: Systemic medication adjustment was associated with short-term CMVR relapse after allo-HSCT. Because treatment changes were clinically indicated and heterogeneous, the findings remain associative, and confounding by indication cannot be excluded.

## 1. Introduction

Cytomegalovirus retinitis (CMVR) is a potentially vision-threatening manifestation of cytomegalovirus infection in immunocompromised patients. After allogeneic hematopoietic stem cell transplantation (allo-HSCT), delayed immune reconstitution, systemic CMV reactivation, graft-versus-host disease (GVHD), and prolonged immunosuppression may contribute to the development or recurrence of CMV disease [1,2,3,4,5]. Although antiviral treatment can control local retinal activity, systemic treatment frequently requires modification because of infection, cytopenia, organ dysfunction, GVHD, or recurrent CMV DNAemia [2,3,4,5,6].

Systemic treatment changes can affect antiviral exposure and the degree of immunosuppression [7]. However, the clinical condition leading to a medication change may also be associated with relapse, which introduces confounding by indication. Evidence specifically examining medication adjustment in relation to subsequent CMVR relapses is limited, particularly in studies that account for correlation between both eyes and include intraocular biomarkers.

This study evaluated the association between systemic medication adjustment and short-term CMVR relapses in a prospective cohort of patients after allo-HSCT. The main objective was to determine whether medication adjustment was associated with relapses after local CMVR control. Aqueous IL-8 and CMV DNA were also examined as inflammatory and virologic markers. IL-8 was prespecified because it formed part of the clinical monitoring protocol and previous studies in HIV-negative and post-HSCT CMVR have supported its use as an adjunct marker of intraocular disease activity and treatment response [8,9]. Previous studies have also characterized aqueous inflammatory profiles in uveitis [10], linked intraocular CMV viral load to ocular disease activity [11], and evaluated standardized CMV-specific immune monitoring for recurrent CMV reactivation after HSCT [12].

## 2. Materials and Methods

### 2.1. Study Cohort, Treatment, and Follow-Up

This prospective cohort study was conducted at the Department of Ophthalmology, Peking University People’s Hospital, from December 2020 through August 2024. Consecutive patients with CMVR after allo-HSCT were enrolled. CMVR was diagnosed on the basis of characteristic funduscopic findings together with an aqueous humor CMV DNA level of at least 1.0 × 10^3^ copies/mL. Patients were eligible if allo-HSCT had been performed within the preceding 12 months, serum HIV testing was negative, and at least three months of follow-up after local treatment were available. Eyes with major coexisting ocular disease or a history of intraocular surgery were excluded.

All patients received systemic ganciclovir or valganciclovir together with intravitreal ganciclovir (3 mg) twice weekly for four doses [13]. Before treatment, aqueous humor was collected for CMV DNA quantification and IL-8 measurement, and peripheral blood was obtained for systemic CMV assessment. IL-8 was included because it was part of the prespecified clinical monitoring protocol rather than selected through post hoc screening of multiple cytokines. Previous CMVR studies reported associations between aqueous IL-8 and intraocular CMV activity and evaluated IL-8 as an adjunct to CMV DNA for treatment monitoring [8,9]. Treatment response was reassessed after the fourth injection. Local treatment was subsequently continued or modified according to the virologic and inflammatory response and was discontinued when aqueous CMV DNA became undetectable (<1.0 × 10^3^ copies/mL) or the IL-8 level was <30 pg/mL. After discontinuation of local treatment, patients were examined every two weeks for three months. Complete resolution was defined clinically by the absence of new or enlarging areas of active retinitis and inactive borders of previously affected lesions on funduscopic examination. Active retinitis was defined by new or enlarging retinal whitening or necrotizing retinitis, with or without hemorrhage, consistent with CMVR. Relapses at the eye level were defined as the reappearance of active retinitis after prior complete resolution, with or without renewed detection of aqueous CMV DNA. Assessments were performed during the scheduled ophthalmic visits according to the same predefined clinical criteria; the protocol standardized the assessment criteria rather than requiring a single examiner for every visit.

### 2.2. Systemic Medication Adjustment

Systemic medication adjustment was defined as a documented initiation, dose escalation, or dose reduction of systemic immunosuppressive, corticosteroid, or anti-infective therapy prompted by a systemic clinical condition after discontinuation of local antiviral treatment. The date of the first systemic medication adjustment after local treatment discontinuation was retrieved from the medical record and used for the timing analyses. Medication class, modification type, clinical indication, and first adjustment date were available for all 18 patients who underwent adjustment. Recorded indications were grouped descriptively as infection or viral reactivation, hematologic or immune abnormality, GVHD-related disease, organ dysfunction or bleeding, and oncologic or transplant-related conditions. Because the individual medication and indication subgroups were small and clinically heterogeneous, they were described but not entered separately into multivariable models.

### 2.3. Statistical Analysis

Continuous variables were summarized as median (range), and categorical variables were summarized as *n* (%). Patient-level characteristics were compared using one record per patient, whereas ocular characteristics were compared at the eye level. The primary outcome was time to CMVR relapse at the eye level. The eye was selected as the principal unit because aqueous CMV DNA, aqueous IL-8, and relapse were assessed separately for each affected eye, and bilateral eyes could differ in biomarker levels and relapse timing. Cox proportional hazards models were fitted with robust standard errors clustered by patient to account for correlation between fellow eyes. The principal multivariable model (Model 1) included systemic medication adjustment and baseline aqueous IL-8. A second model included medication adjustment and baseline aqueous CMV DNA. Proportional hazards assumptions were assessed using Schoenfeld residuals. Because baseline aqueous CMV DNA did not satisfy this assumption, an extended Cox model including an interaction between CMV DNA and log(time) was fitted.

Sensitivity analyses included a single-eye Cox analysis—in which the right eye was retained when both eyes were affected—patient-level logistic regression with relapses defined as the first recurrence in either eye, and patient-grouped 10-fold cross-validated LASSO Cox regression. To account explicitly for the timing of systemic medication adjustment, an additional start-stop time-dependent Cox model was fitted at the eye level, with adjustment status coded as 0 before the first adjustment date and 1 thereafter; robust standard errors were clustered by patient. A patient-level Cox model using time to the first relapse in either eye was also fitted, together with a patient-level time-dependent Cox sensitivity analysis. Patient-level relapse-free survival was estimated using the Kaplan–Meier method and compared descriptively with the log-rank test. Time-dependent AUCs were calculated in the independent single-eye dataset as an exploratory assessment of model discrimination. All tests were two-sided, and *p* < 0.05 was considered statistically significant. The original analyses were performed in R software (version 4.3.1) within RStudio (version 2026.01.2+418), and the added time-dependent Cox analyses were reproduced using Python 3.13.5 with statsmodels 0.14.6.

### 2.4. Missing Data

There were no missing values for baseline aqueous CMV DNA, baseline aqueous IL-8, systemic medication-adjustment status, relapse status, or follow-up time in the analytic cohort. For all 18 patients who underwent systemic medication adjustment, the date of the first adjustment, medication class, modification type, and clinical indication were available. Exact dose quantities were not uniformly available and were not included in the inferential analyses. No statistical imputation was performed.

## 3. Results

### 3.1. Cohort and Medication-Adjustment Indications

The cohort included 44 patients (65 eyes), comprising 25 men (56.8%) and 19 women, with a mean age of 33.5 ± 12.9 years. During the three-month follow-up period, 24 eyes (36.9%) from 19 patients (43.2%) relapsed. Systemic medication adjustment was recorded in 18 patients (40.9%). Among the 19 patients with relapses, 13 (68.4%) underwent medication adjustment, compared with 5 of 25 patients without relapses (20.0%; *p* = 0.001). The most frequent indication was infection or viral reactivation (6/18, 33.3%), followed by hematologic or immune abnormalities (4/18, 22.2%) and GVHD-related conditions (4/18, 22.2%) (Table 1). Exact timing of the first systemic medication adjustment was available for all 18 adjusted patients. The first adjustment occurred 25.5 days (median) after discontinuation of local antiviral therapy (range, 7 to 72 days). Among the 13 adjusted patients who subsequently relapsed, all first adjustments preceded relapses, with a median interval of 15 days from adjustment to relapse (range, 3 to 52 days). Patient-level timing and medication details are provided in Supplementary Table S6.

Compared with patients without relapse, those with relapse were older (median, 39 vs. 28 years; *p* = 0.012), had a higher peak blood CMV load (4.68 vs. 2.74 × 10^4^ copies/mL; *p* = 0.025), and had later detection of blood CMV after transplantation (83 vs. 58 days; *p* = 0.048). At the eye level, eyes that relapsed had higher baseline aqueous CMV DNA levels (5.14 vs. 0.08 × 10^5^ copies/mL; *p* < 0.001), received more intravitreal injections (8 vs. 7; *p* = 0.019), and had worse baseline and terminal visual acuity (*p* = 0.009 and *p* = 0.006, respectively). Complete baseline comparisons are presented in Supplementary Table S1.

### 3.2. Primary and Sensitivity Analyses

In Model 1, systemic medication adjustment was associated with relapses (HR, 8.254; 95% CI, 2.656 to 25.645; *p* < 0.001). Baseline aqueous IL-8 was also associated with relapses (HR, 1.190 per 100 pg/mL; 95% CI, 1.088 to 1.301; *p* < 0.001). Model 1 satisfied the proportional hazards assumption (global *p* = 0.420). In the conventional CMV DNA model, medication adjustment remained associated with relapse (HR, 5.569; 95% CI, 2.364 to 13.119; *p* < 0.001), whereas baseline aqueous CMV DNA did not satisfy the proportional hazards assumption (*p* = 0.001; global *p* = 0.003). In the extended model, medication adjustment remained associated with relapses (HR, 4.735; 95% CI, 1.988 to 11.279; *p* < 0.001), and the estimated association for CMV DNA varied over time (Supplementary Table S3).

The association between medication adjustment and relapse was also observed in the independent single-eye analyses (Model 1: HR, 6.004; 95% CI, 2.137 to 16.869; *p* < 0.001; Model 2: HR, 4.221; 95% CI, 1.563 to 11.400; *p* = 0.004) and in the patient-level logistic regression (OR, 8.667; 95% CI, 2.187 to 34.346; *p* = 0.002). In the added eye-level time-dependent Model 1, systemic medication adjustment remained associated with relapse (HR, 11.360; 95% CI, 4.330 to 29.803; *p* < 0.001), and aqueous IL-8 remained associated with relapse (HR, 1.162 per 100 pg/mL; 95% CI, 1.060 to 1.274; *p* = 0.001). In the patient-level Cox analysis using time to first relapse in either eye, medication adjustment was associated with relapses (HR, 4.477; 95% CI, 1.688 to 11.878; *p* = 0.003). The corresponding patient-level time-dependent Cox analysis yielded an HR of 7.206 (95% CI, 2.791 to 18.606; *p* < 0.001). These primary and sensitivity analyses are summarized in Table 2. Under the more conservative lambda.1se LASSO criterion, systemic medication adjustment and baseline aqueous CMV DNA were retained in the model (Supplementary Table S5 and Supplementary Figure S4).

### 3.3. Patient-Level Relapse-Free Survival and Exploratory Discrimination

Patient-level relapse-free survival rates differ according to eventual systemic medication adjustment status (descriptive log-rank *p* < 0.001; Figure 1). During follow-up, 18 patients underwent adjustment, and 26 did not. Because medication adjustment occurred after baseline in the exposed patients, Figure 1 is presented as a descriptive display of eventual adjustment status; temporal inference was based on the time-dependent Cox analyses. In the exploratory time-dependent AUC analysis of the 44-patient single-eye dataset, AUCs ranged from 0.729 to 0.777 for Model 1 and from 0.733 to 0.846 for Model 2 at days 37, 43, and 58. No comparison between the two models reached statistical significance (all *p* ≥ 0.091; Supplementary Table S4 and Supplementary Figures S2 and S3).

## 4. Discussion

In this prospective cohort, systemic medication adjustment after local CMVR control was associated with short-term relapses in patients who had undergone allo-HSCT. The association was observed in the primary patient-clustered eye-level analysis and remained directionally consistent in independent single-eye, patient-level time-to-first-relapse, and time-dependent Cox analyses. The eye-level approach was retained as the principal analysis because aqueous biomarkers and relapse were eye-specific, whereas the patient-level analyses addressed the alternative unit of analysis requested for a patient-level exposure. The availability of exact first-adjustment dates also allowed the temporal sensitivity analysis to distinguish person-time before and after systemic medication adjustment.

The observed association should not be interpreted as a treatment effect. Medication changes after transplantation occurred for heterogeneous clinical reasons. In this cohort, infection or viral reactivation was the most frequent indication, followed by hematologic or immune abnormalities and GVHD-related conditions. Different modifications are not clinically equivalent. Initiation or escalation of glucocorticoids or other immunosuppressive therapy may have different effects on host antiviral immunity from dose reduction of immunosuppression or initiation of anti-infective treatment. The subgroup sizes were too small to estimate these effects separately. Confounding by indication is therefore particularly important: the infection, immune abnormality, GVHD, organ dysfunction, or other conditions that prompted a medication change may itself be associated with CMVR relapses. The observed association may reflect both the treatment modification and the underlying clinical condition, and these effects cannot be separated in the present study.

Aqueous biomarkers provided additional information. IL-8 is a proinflammatory chemokine relevant to intraocular inflammation [10]. It was selected a priori because it was incorporated into the clinical monitoring protocol and prior studies in HIV-negative and post-HSCT CMVR reported that aqueous IL-8 tracks intraocular CMV activity and can complement aqueous CMV DNA when assessing treatment response [8,9]. In the present cohort, baseline aqueous IL-8 was associated with time to relapse in the primary and time-dependent Model 1 analyses. Baseline aqueous CMV DNA was also associated with relapse, consistent with previous evidence linking intraocular CMV burden to ocular disease activity [11]. The positive CMV DNA × log(time) interaction indicates that the association between the baseline viral burden and subsequent relapse hazard was not constant during follow-up. The estimated HR per 10^4^ copies/mL increased from 1.012 on day 30 to 1.033 (day 45), 1.048 (day 60), and 1.069 (day 90). This does not indicate that the baseline viral load itself increased over time; rather, the association of the baseline measurement with subsequent relapse differed according to follow-up time. The biological basis for this pattern cannot be determined from the present observational data.

Medication adjustment may therefore identify a period in which closer ophthalmic follow-up could be considered after local CMVR control. The first systemic medication adjustment occurred a median of 25.5 days after local treatment discontinuation, and among adjusted patients who later relapsed, the adjustment preceded relapse in all cases, with a median interval of 15 days. However, this temporal sequence does not establish causality. This study did not evaluate the effects of intensified surveillance or prophylactic antiviral treatment, and no specific management strategy can be recommended from these data.

This study has several limitations. It was conducted at a single center and included 44 patients, which resulted in wide confidence intervals for several estimates. Although the date of the first systemic medication adjustment, medication class, modification type, and clinical indication were available for all 18 adjusted patients, exact dose quantities were not uniformly available. Medication adjustments were clinically indicated rather than assigned independently of the patient’s condition, making confounding by indication a central limitation. The medication and indication subgroups were small and heterogeneous, so separate drug-specific or indication-specific effect estimates were not considered reliable. Residual confounding could not be excluded. The exploratory time-dependent AUC analysis was not externally validated and should not be interpreted as a clinically applicable prediction model. The time-dependent analyses strengthen the temporal characterization of the association but do not eliminate the limitations of the observational design.

## 5. Conclusions

Systemic medication adjustment was associated with short-term CMVR relapse after allo-HSCT, and the association remained present in analyses that incorporated the timing of the first medication change and in patient-level time-to-first-relapse analysis. Because systemic treatment changes were clinically indicated and heterogeneous, these findings should be interpreted as associative and exploratory rather than causal. Larger prospective studies are needed to distinguish the effects of specific medication changes from the underlying clinical conditions that prompted them.

## Figures and Tables

**Figure 1:**
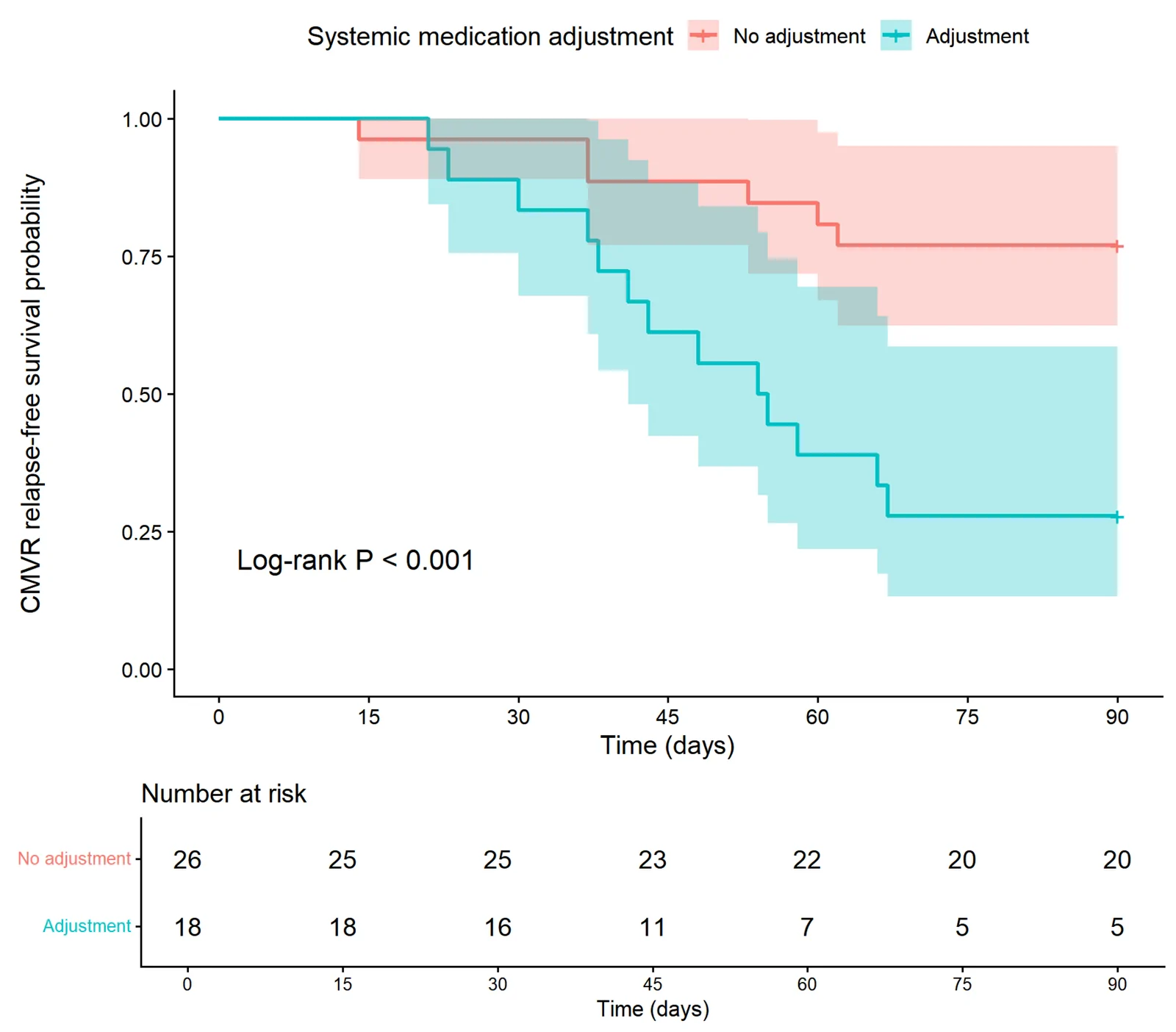
Patient-level Kaplan–Meier curves for CMVR relapse-free survival according to eventual systemic medication adjustment status. The event was defined as the first relapse in either eye. The number-at-risk table is displayed directly below the curve. Shaded bands indicate 95% confidence intervals, and tick marks indicate censored observations. Because medication adjustment occurred during follow-up, the figure is descriptive; temporal associations were evaluated using time-dependent Cox models. Descriptive log-rank *p* < 0.001.

**Table 1:** Recorded indications for systemic medication adjustment (n = 18 patients).

Clinical Category	Total, n (%)	Relapse, n	No Relapse, n
Infection or viral reactivation	6 (33.3%)	4	2
Hematologic or immune abnormality	4 (22.2%)	4	0
GVHD-related	4 (22.2%)	2	2
Organ dysfunction or bleeding	2 (11.1%)	1	1
Oncologic or transplant-related	2 (11.1%)	2	0

Note: Categories are presented descriptively. Relapse status was defined at the patient level as the first relapse in either eye. Medication class, modification type, clinical indication, and the date of the first adjustment were available for all 18 adjusted patients. Exact dose quantities were not modeled. Detailed timing and medication characteristics are provided in Supplementary Table S6.

**Table 2:** Primary multivariable and sensitivity analyses.

Analysis	Variable	Effect Estimate (95% CI)	*p*
Primary clustered Cox, Model 1	Systemic medication adjustment	HR 8.254 (2.656–25.645)	<0.001
Primary clustered Cox, Model 1	Aqueous IL-8, per 100 pg/mL	HR 1.190 (1.088–1.301)	<0.001
Conventional clustered Cox, Model 2	Systemic medication adjustment	HR 5.569 (2.364–13.119)	<0.001
Conventional clustered Cox, Model 2	Aqueous CMV DNA, per 10^4^ copies/mL	HR 1.022 (1.013–1.032)	<0.001
Extended Cox, Model 2	Systemic medication adjustment	HR 4.735 (1.988–11.279)	<0.001
Extended Cox, Model 2	CMV DNA × log(time)	HR 1.051 (1.022–1.082)	<0.001
Single-eye Cox, Model 1	Systemic medication adjustment	HR 6.004 (2.137–16.869)	<0.001
Single-eye Cox, Model 2	Systemic medication adjustment	HR 4.221 (1.563–11.400)	0.004
Patient-level logistic regression	Systemic medication adjustment	OR 8.667 (2.187–34.346)	0.002
Time-dependent clustered Cox, Model 1	Medication adjustment, time-dependent	HR 11.360 (4.330–29.803)	<0.001
Time-dependent clustered Cox, Model 1	Aqueous IL-8, per 100 pg/mL	HR 1.162 (1.060–1.274)	0.001
Patient-level time-to-first-relapse Cox	Systemic medication adjustment	HR 4.477 (1.688–11.878)	0.003
Patient-level time-dependent Cox	Medication adjustment, time-dependent	HR 7.206 (2.791–18.606)	<0.001

Note: The primary Cox models included all 65 eyes and used robust standard errors clustered by patient (24 eye-level relapse events). The single-eye Cox models included 44 eyes and 19 events. The time-dependent eye-level Cox analysis used start-stop intervals, with systemic medication adjustment coded as 0 before the first adjustment date and 1 thereafter, and robust standard errors clustered by patient. Patient-level Cox analyses included 44 patients and 19 first-relapse events. Model 2 is presented for completeness. Because baseline aqueous CMV DNA did not satisfy the proportional hazards assumption in the conventional model, the extended model was used for interpretation. HR, hazard ratio; OR, odds ratio; CI, confidence interval.

## Data Availability

The data presented in this study are available from the corresponding author upon reasonable request. The data are not publicly available due to patient privacy and ethical restrictions.

## Supplementary Materials

The following supporting information can be downloaded at https://www.mdpi.com/article/10.3390/biomedicines14102189/s1. Figure S1: Treatment and monitoring algorithm for patients with CMVR; Table S1: Baseline characteristics according to CMVR relapse status; Table S2: Univariate Cox regression of factors associated with CMVR relapse at the eye level; Table S3: Extended Model 2 with a time-varying effect of baseline aqueous CMV DNA; Table S4: Time-dependent AUC comparison in the single-eye complete-case analysis; Figure S2: Time-dependent receiver operating characteristic curves for Model 1 and Model 2 at days 37, 43, and 58 in the single-eye complete-case analysis; Figure S3: Time-dependent AUCs and 95% confidence intervals for Model 1 and Model 2 at days 37, 43, and 58; Table S5: Nonzero coefficients from patient-grouped LASSO Cox regression; Figure S4: Ten-fold patient-grouped cross-validation curve for LASSO Cox regression; Table S6: Timing and characteristics of systemic medication adjustments.
