# Supplementary material for: Systemic Medication Adjustment and Short-Term Relapse of Cytomegalovirus Retinitis After Allogeneic Hematopoietic Stem Cell Transplantation: A Research Communication
Source: Biomedicines. 2026 Sep 28;14(10):2189. doi: 10.3390/biomedicines14102189 (PMC13701335; doi:10.3390/biomedicines14102189)
Supplement: Supplementary file 1 [file biomedicines-14-02189-s001.zip › biomedicines-4562547-supplementary.pdf]

Supplementary Material

The Supplementary Material contains the full descriptive and exploratory analyses that were omitted from the main Research Communication to maintain a concise presentation of the primary findings.

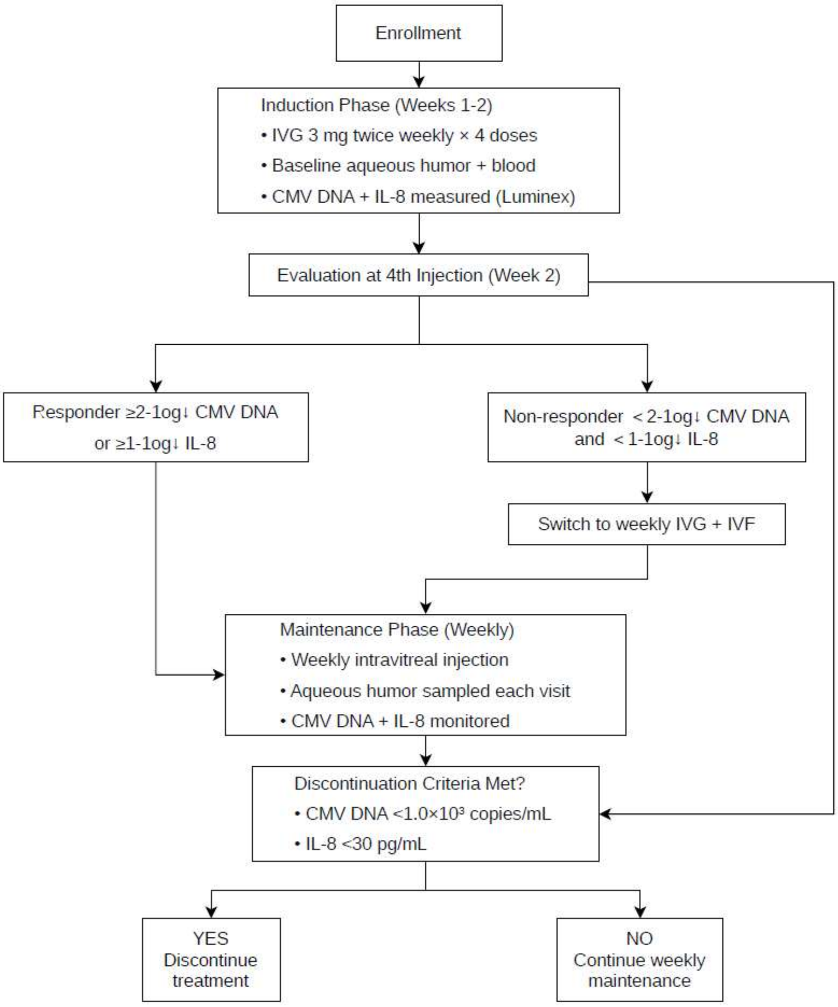

Supplementary Figure S1. Treatment and monitoring algorithm for patients with CMVR. IVG, intravitreal ganciclovir; IVF, intravitreal foscarnet; IL-8, interleukin-8; CMV, cytomegalovirus.

Supplementary Table S1. Baseline characteristics according to CMVR relapse status

Level	Variable	Relapse group	Non-relapse group	*P*
Patient	Age, years	39.00 (15.00-70.00)	28.00 (18.00-49.00)	0.012
Patient	Blood CMV peak, x10^4 copies/mL	4.68 (1.10-31.90)	2.74 (0.38-16.00)	0.025
Patient	Time from transplantation to CMVR diagnosis, days	163.00 (24.00-520.00)	143.00 (87.00-342.00)	0.644
Patient	Blood CMV detection after transplantation, days	83.00 (37.00-191.00)	58.00 (19.00-285.00)	0.048
Patient	Male sex, n (%)	13 (68.4%)	12 (48.0%)	0.176
Patient	Systemic medication adjustment, n (%)	13 (68.4%)	5 (20.0%)	0.001
Patient	Baseline blood CMV positive, n (%)	8 (42.1%)	4 (16.0%)	0.054
Eye	Baseline aqueous CMV DNA, x10^5 copies/mL	5.14 (0.00-20.60)	0.08 (0.00-4.82)	<0.001
Eye	Baseline aqueous IL-8, pg/mL	106.17 (21.11-1840.10)	110.80 (2.18-1409.70)	0.262

Level	Variable	Relapse group	Non-relapse group	*P*
Eye	Number of intravitreal injections	8.00 (4.00-14.00)	7.00 (4.00-16.00)	0.019
Eye	Baseline visual acuity, logMAR	0.80 (0.00-3.00)	0.30 (0.00-2.60)	0.009
Eye	Terminal visual acuity, logMAR	0.55 (0.20-3.00)	0.30 (0.00-2.60)	0.006
Eye	Baseline IOP, mmHg	15.00 (10.00-21.00)	15.00 (11.00-29.00)	0.613
Eye	Anterior chamber inflammation, n (%)	11 (45.8%)	22 (53.7%)	0.543
Eye	Macular involvement, n (%)	14 (58.3%)	25 (61.0%)	0.834
Eye	Involvement of >=3 quadrants, n (%)	12 (50.0%)	14 (34.1%)	0.208

Patient-level denominators were 19 patients with relapse and 25 patients without relapse. Eye-level denominators were 24 eyes with relapse and 41 eyes without relapse. Continuous variables are presented as median (range), and categorical variables as n (%).

Supplementary Table S2. Univariate Cox regression of factors associated with CMVR relapse at the eye level

Variable	HR (95% CI)	*P*
Age, per 1-year increase	1.050 (1.020-1.082)	0.001
Sex, male vs female	1.965 (0.692-5.584)	0.205
Systemic medication adjustment, yes vs no	6.019 (2.110-17.175)	<0.001
Blood CMV detection after transplantation, per day	1.001 (0.993-1.009)	0.811
Blood CMV peak, per 10^5 copies/mL	1.443 (1.099-1.895)	0.008
Time from transplantation to CMVR diagnosis, per day	1.001 (0.995-1.006)	0.829
Baseline blood CMV positive vs negative	2.228 (0.829-5.989)	0.112
Baseline aqueous CMV DNA, per 10^4 copies/mL	1.020 (1.011-1.029)	<0.001
Baseline aqueous IL-8, per 100 pg/mL	1.132 (1.033-1.241)	0.008
Intravitreal injections, per injection	1.163 (0.995-1.358)	0.057
Baseline visual acuity, per 0.1 logMAR	1.054 (0.999-1.112)	0.057
Terminal visual acuity, per 0.1 logMAR	1.045 (1.004-1.087)	0.032
Baseline IOP, per 1 mmHg	0.944 (0.838-1.065)	0.351
Involvement of >=3 quadrants, yes vs no	1.509 (0.645-3.533)	0.343
Macular involvement, yes vs no	1.004 (0.428-2.355)	0.993
Anterior chamber inflammation, yes vs no	0.771 (0.303-1.963)	0.585

Models included all 65 eyes and used robust standard errors clustered by patient (24 relapse events at the eye level).

Supplementary Table S3. Extended Model 2 with a time-varying effect of baseline aqueous CMV DNA

Term or time point	HR	95% CI	*P*
Systemic medication adjustment	4.735	1.988-11.279	<0.001

Term or time point	HR	95% CI	*P*
CMV DNA main term	0.854	0.768-0.949	0.003
CMV DNA x log(time)	1.051	1.022-1.082	<0.001
Time-specific CMV DNA effect: Day 30	1.012	0.999-1.025	-
Time-specific CMV DNA effect: Day 45	1.033	1.021-1.044	-
Time-specific CMV DNA effect: Day 60	1.048	1.031-1.064	-
Time-specific CMV DNA effect: Day 90	1.069	1.041-1.097	-

The model included systemic medication adjustment, baseline aqueous CMV DNA per 10^4 copies/mL, and an interaction between CMV DNA and log(time). Because the main CMV DNA term is conditional on log(time) = 0, time-specific HRs are more informative for interpretation.

Supplementary Table S4. Time-dependent AUC comparison in the single-eye complete-case analysis

Time point, days	Model 1 AUC (95% CI)	Model 2 AUC (95% CI)	*P* for comparison
37	0.777 (0.408-1.000)	0.733 (0.485-0.981)	0.681
43	0.729 (0.503-0.955)	0.753 (0.540-0.967)	0.710
58	0.761 (0.593-0.929)	0.846 (0.697-0.995)	0.091

Model 1 included systemic medication adjustment and baseline aqueous IL-8. Model 2 included systemic medication adjustment and baseline aqueous CMV DNA. The analysis included 44 independent eyes and 19 relapse events. *P* values were obtained using the iid representation based comparison procedure implemented in timeROC.

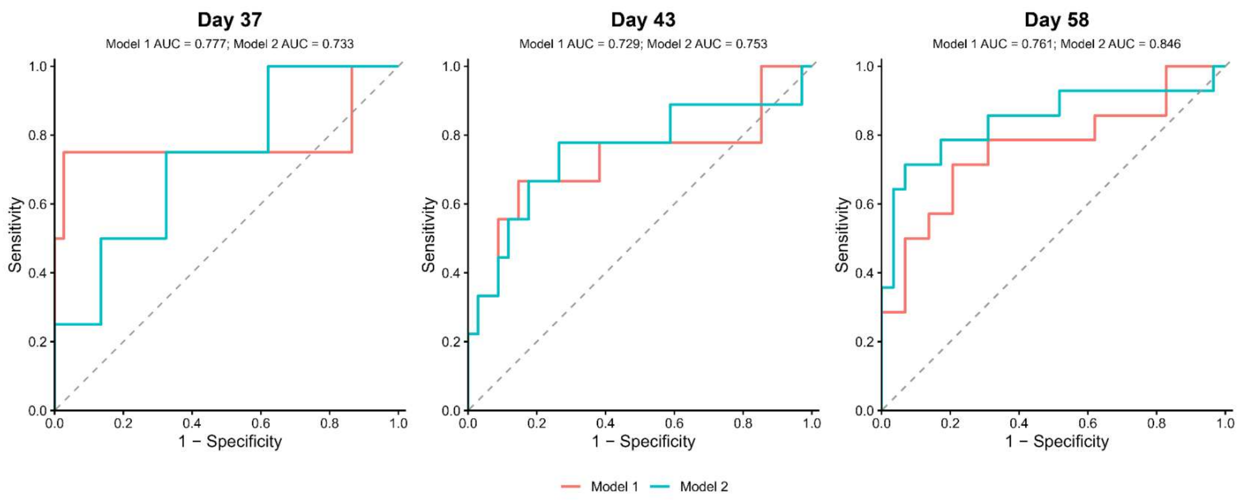

Supplementary Figure S2. Time-dependent receiver operating characteristic curves for Model 1 and Model 2 at days 37, 43, and 58 in the single-eye complete-case analysis.

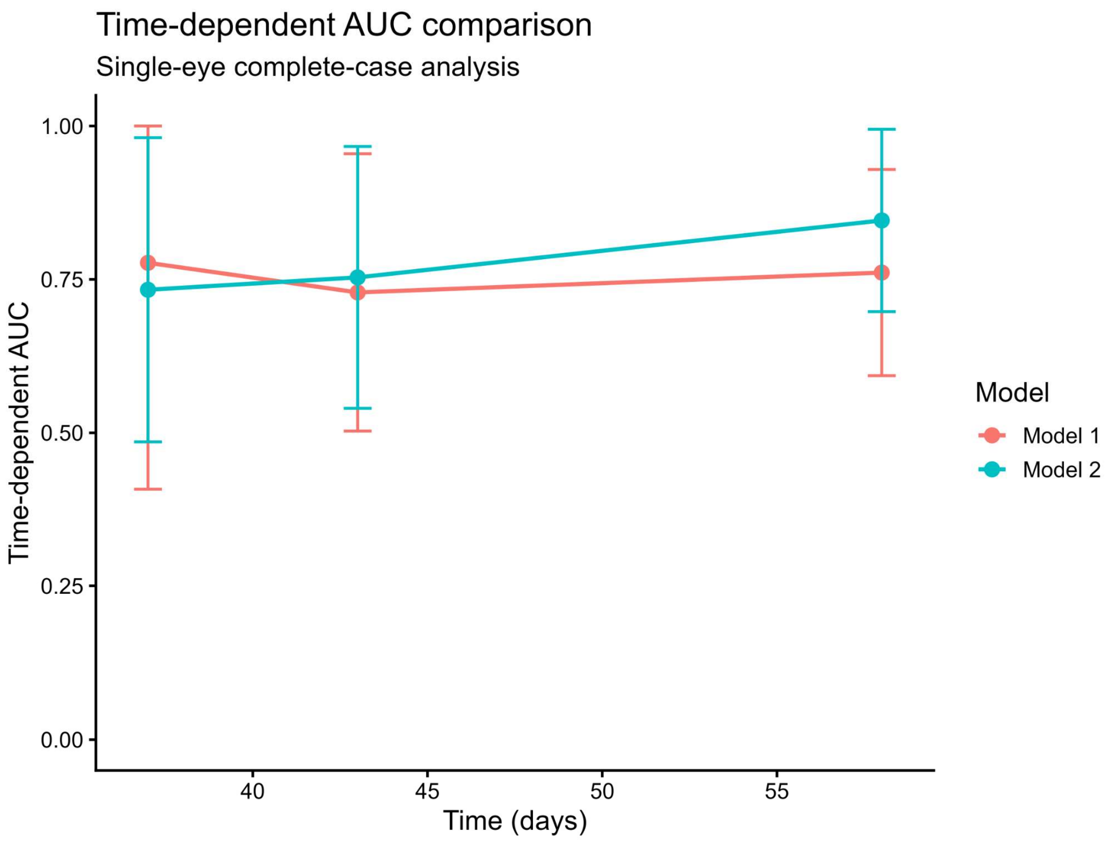

Supplementary Figure S3. Time-dependent AUCs and 95% confidence intervals for Model 1 and Model 2 at days 37, 43, and 58. No comparison between the models reached statistical significance.

Supplementary Table S5. Nonzero coefficients from patient-grouped LASSO Cox regression

Criterion	Variable	Coefficient	Penalized HR
lambda.min	Systemic medication adjustment	1.2082	3.348
lambda.min	Blood CMV peak, per 10^5 copies/mL	0.0826	1.086
lambda.min	Baseline aqueous IL-8, per 100 pg/mL	0.0667	1.069
lambda.min	Number of intravitreal injections	0.0467	1.048
lambda.min	Baseline IOP	-0.0356	0.965
lambda.min	Terminal visual acuity, per 0.1 logMAR	0.0148	1.015
lambda.min	Baseline aqueous CMV DNA, per 10^4 copies/mL	0.0143	1.014
lambda.min	Age	0.0080	1.008
lambda.min	Baseline visual acuity, per 0.1 logMAR	0.0051	1.005
lambda.1se	Systemic medication adjustment	0.2254	1.253
lambda.1se	Baseline aqueous CMV DNA, per 10^4 copies/mL	0.0093	1.009

The analysis included 65 eyes, 24 relapse events at the eye level, and 44 patients. Ten-fold cross-validation was grouped by patient. Penalized HRs are shrunken estimates and should not be compared directly with HRs from conventional Cox models.

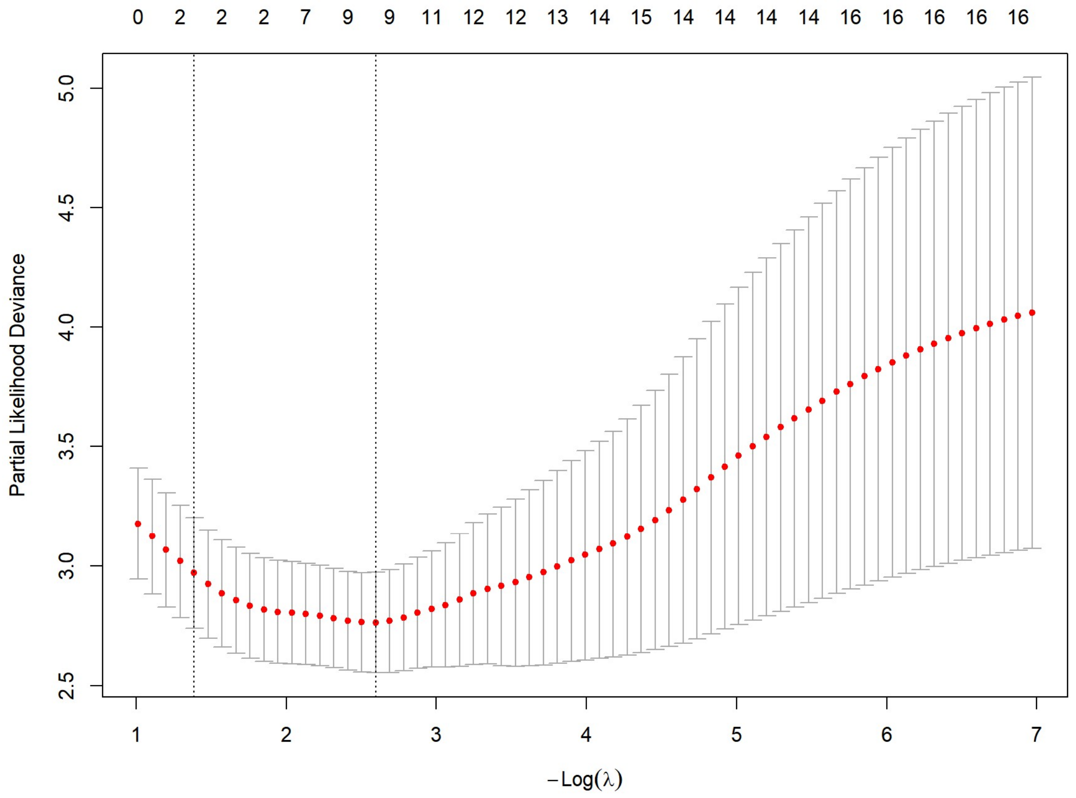

Supplementary Figure S4. Ten-fold patient-grouped cross-validation curve for LASSO Cox regression. The vertical dotted lines indicate lambda.min and lambda.1se, and the numbers above the curve indicate the number of nonzero coefficients.

Supplementary Table S6. Timing and characteristics of systemic medication adjustments

Medication adjustment is presented once per patient. Timing is expressed relative to discontinuation of local antiviral therapy. The final interval is measured to relapse for patients who relapsed and to censoring for patients without relapse.

Patient ID	Medication class	Modification type	Clinical indication	Clinical category	Days to first adjustment	Outcome	Days from adjustment to endpoint
1	Glucocorticoid	Initiation	Severe pneumonia	Infection or viral reactivation	29	Censored	61
5	Glucocorticoid and Cyclosporine	Dose escalation	Hepatic GVHD	GVHD-related	72	Censored	18
12	Antibacterial therapy	Initiation	Systemic bacterial infection	Infection or viral reactivation	7	Censored	83
17	Glucocorticoid	Dose escalation	Immune-mediated cytopenia	Hematologic or immune abnormality	14	Relapse	16
20	Glucocorticoid	Dose escalation	Chronic oral GVHD	GVHD-related	32	Censored	58
23	Cyclosporine	Initiation	Immune-mediated thrombocytopenia	Hematologic or immune abnormality	29	Relapse	9
25	Immunosuppressant	Dose reduction	Renal dysfunction	Organ dysfunction or bleeding	56	Censored	34
27	Glucocorticoid and Cyclosporine	Dose escalation	Cutaneous GVHD	GVHD-related	9	Relapse	12
29	Glucocorticoid and Methotrexate	Initiation	Central nervous system leukemia	Oncologic or transplant-related	14	Relapse	52
32	Glucocorticoid and Co-trimoxazole	Initiation	Moderate-to-severe PJP	Infection or viral reactivation	11	Relapse	12
34	Immunosuppressant	Dose reduction	Hemorrhagic cystitis	Organ dysfunction or bleeding	33	Relapse	15
36	Glucocorticoid and Cyclosporine	Dose escalation	Gastrointestinal GVHD	GVHD-related	38	Relapse	3
37	Glucocorticoid	Dose reduction	Preparation for second allo-HSCT	Oncologic or transplant-related	23	Relapse	35
38	Glucocorticoid and Cyclosporine	Dose reduction	Severe lymphopenia	Hematologic or immune abnormality	14	Relapse	41
39	Immunosuppressant	Dose reduction	CMV reactivation	Infection or viral reactivation	29	Relapse	14
42	Glucocorticoid	Dose reduction	Fungal infection	Infection or viral reactivation	23	Relapse	31
43	Glucocorticoid	Dose reduction	Fungal pneumonia	Infection or viral reactivation	27	Relapse	40
44	Glucocorticoid	Dose reduction	Hypogammaglobulinemia	Hematologic or immune abnormality	24	Relapse	13
